# Pyrazinamide related prolonged drug-induced liver injury: A case report

**DOI:** 10.1097/MD.0000000000030955

**Published:** 2022-09-30

**Authors:** Yeh-Chin Wang, Kai-Hsiang Chen, Yen-Lin Chen, Shu-Wen Lin, Wang-Da Liu, Jann-Tay Wang, Chien-Ching Hung

**Affiliations:** a Department of Internal Medicine, National Taiwan University Hospital and National Taiwan University College of Medicine, Taipei, Taiwan; b Department of Pharmacy, National Taiwan University Hospital, Taipei, Taiwan; c Graduate Institute of Clinical Pharmacy, National Taiwan University, Taipei, Taiwan; d Department of Medicine, National Taiwan University Cancer Center, Taipei, Taiwan; e Department of Internal Medicine, National Taiwan University Hospital Yun-Lin Branch, Yun-Lin County, Taiwan; f Department of Tropical Medicine and Parasitology, National Taiwan University College of Medicine, Taipei, Taiwan; g Department of Medical Research, China Medical University Hospital, Taichung, Taiwan; h China Medical University, Taichung, Taiwan.

**Keywords:** hepatotoxicity, isoniazid, NAT2 polymorphism, pharmacogenetic, pyrazinamide

## Abstract

Drug induced liver injury (DILI) is a common side effect causing treatment discontinuation during tuberculosis (TB) treatment, and pyrazinamide (PZA) usually leads to a delayed and prolonged abnormal liver function of the 4 standard anti-tuberculosis regimens. However, a prolonged hepatitis lasting more than 4 months is rarely reported.

**Patient concerns:**

A 78-year-old man presented with general weakness and poor appetite on his seventh week of anti-TB treatment for tuberculosis lymphadenitis.

**Diagnosis:**

Drug induced liver injury, PZA-related. NAT2 slow acetylator phenotype was accidentally found during workup of DILI.

**Intervention:**

A liver biopsy was performed and PZA-related DILI was suspected. All anti-TB medications were therefore discontinued.

**Outcome:**

After withholding all anti-TB medications for 4 months, the elevations of aminotransferases and hyperbilirubinemia completely resolved. Anti-TB therapy was switched to ethambutol and levofloxacin for 15 months without adverse events. Long-term ultrasound follow-up was performed and cervical lymphadenopathy completely resolved.

**Conclusion:**

Our patient presents with PZA related prolonged DILI resolved after drug discontinuation for 4 months. NAT2 slow acetylator phenotype may be related to this condition through unknown mechanisms.

## 1. Introduction

Until now, a 6-month, 4-drug regimen remains a standard therapy for drug-susceptible tuberculosis (TB). However, drug-induced liver injury (DILI) is a common cause associated with drug interruption. Host factors including older age, female gender, malnutrition, alcoholism, presence of viral hepatitis and human immunodeficiency virus were associated with higher risk of developing DILI.^[1]^ In addition, patients with N-acetyltransferase 2 (NAT2) slow acetylator phenotype or homozygous wild genotype CYP2E1 c1/c1 are at risk for prolonged hepatitis because of slow metabolization of isoniazid (INH).^[2–4]^ Pyrazinamide (PZA) is another common cause of DILI due to a longer half-life compared with INH and rifampin (RMP).^[1,5]^ Here we present a case of prolonged PZA related DILI after treatment for tuberculous lymphadenitis.

## 2. Case report

This 78-year-old man with underlying type 2 diabetes mellitus had been in his usual state of health until 2 months prior to this admission, when intermittent fever and a mass at his right neck developed. He reported a temperature of 38.5 degrees Celsius without chills, with a frequency of twice every week. There was no consciousness disturbance, swallowing difficulty, palpitations, productive cough, tremor, abdominal discomfort, diarrhea or dysuria, nor was there significant weight loss in the last 6 months or night sweats. One month later, the fevers became more frequent, and the neck mass enlarged with severe pain, but with no wound.

Three days before this admission, he was seen at the Department of Emergency at this hospital due to fever and worsening pain of his neck mass. On examination, the temperature was 39.2 °C, the pulse 98 beats per minute, the respiratory rate 20 breaths per minute, the blood pressure 168/66 mm Hg, and the oxygen saturation 99% while the patient was breathing ambient air. The weight was 62 kg and the height 161 cm, with the body-mass index of 23.9 kg/m^2^. Physical examination revealed a soft, movable and painful lump with irregular margin measuring 2 centimeters in the greatest diameter of level IV of the right neck. There was no oral thrush or neck stiffness.

The white-cell count was 5870 per cubic millimeter, with 69% neutrophils, 20.3% lymphocytes and 1.7% eosinophils; the hemoglobin level was 11.8 g per deciliter, the mean corpuscular volume 81 fl. The platelet count was 332,000 per cubic millimeter. The serum creatinine was 1.3 mg per deciliter, alanine aminotransferase 13 U/L (reference range, 0 –41). The C-reactive protein level was 4.1 microgram per deciliter and the procalcitonin level was 3.94 nanogram per milliliter (reference range < 0.5). The remainder of the metabolic profile was normal.

**Figure 1:**
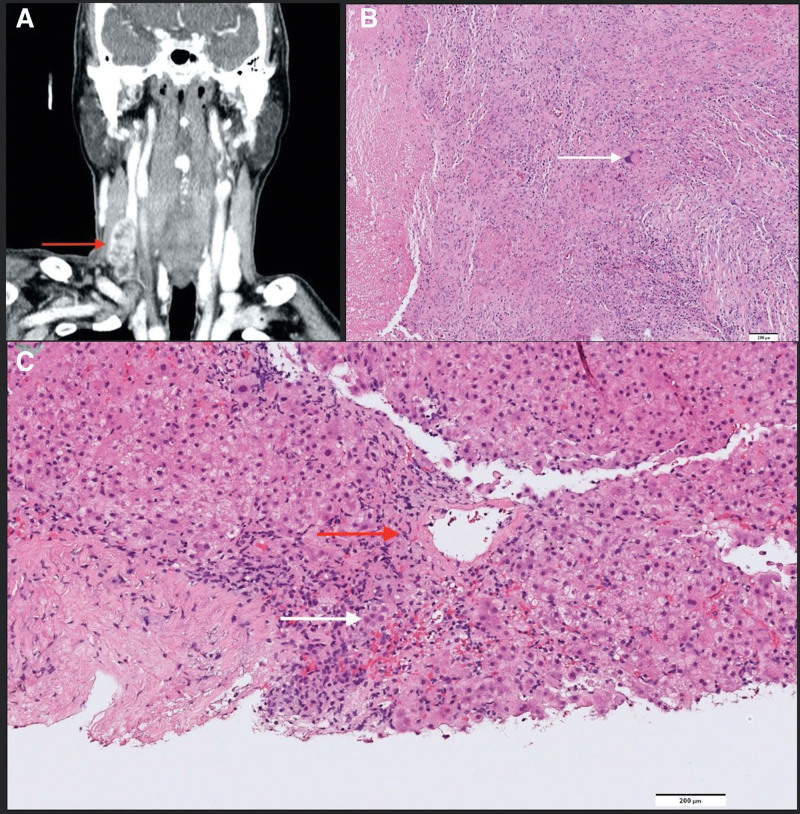
(A) Computed tomography of the head and neck revealing multiple necrotic lymphadenopathy at the right neck (red arrow). (B) Pathology of lymph node biopsy revealed multinucleated giant cell (white arrow) and caseating granulomatous inflammation. (C) Active hepatitis characteristics including portal inflammation, portal zone necrosis (red arrow) and rosette formation (white arrow). There was no caseating granulomatous inflammation nor plasma cell cluster.

A computed tomography (CT) revealed multiple necrotic lymph nodes at his right neck (Fig. 1A) and excisional lymph node biopsy was performed, which disclosed caseating granulomatous inflammation with acid-fast stain positive bacilli (Fig. 1B). The tissue culture subsequently yielded *Mycobacterium tuberculosis*, that was susceptible to all anti-TB agents tested. A standard 4-drug regimen consisting of INH (5 mg/kg), RMP (10 mg/kg), PZA (25 mg/kg) and ethambutol (EMB) (15 mg/kg) was initiated. However, itchy macules over the trunk and extremities developed 7 days after initiation of anti-TB therapy. Two weeks later, the patient was discharged with the regimen of rifabutin (300 mg/day), PZA and levofloxacin (750 mg/day) because of drug eruption on rechallenge with INH and RMP.

Six weeks later, the patient reported general weakness and poor appetite during an outpatient visit. Blood examinations showed elevated liver function, with aspartate aminotransferase (AST) 562 U/L, alanine aminotransferase (ALT) 755 U/L, alkaline phosphatase 441 U/L, gamma glutamyl transferase 760 U/L, total bilirubin 3.43 mg/dL and direct bilirubin 2.47 mg/dL. Tests for viral hepatitis, Epstein-Barr virus, human immunodeficiency virus, autoimmune hepatitis and Wilson’s disease revealed negative results, while follow-up CT was unchanged. The patients reported no exposure to chemicals or consumption of alcohol. A diagnosis of DILI related to anti-TB therapy was made.

Nevertheless, liver enzymes remained high with AST 235 U/L and ALT 385 U/L despite interruption of anti-TB medication for 3 months. A liver biopsy was performed, and the pathology revealed active hepatitis without caseating granulomatous inflammation nor clusters of plasma cells, which argued against the consideration of hepatic tuberculosis and autoimmune hepatitis (Fig. 1C). NAT2 acetylator genotype was determined, which showed NAT2 *5/*6 suggesting slow acetylator phenotype. After withholding all anti-TB medications for 4 months, the elevations of aminotransferases and hyperbilirubinemia completely resolved. Anti-TB therapy was switched to EMB and levofloxacin for 15 months without adverse events. Long-term ultrasound follow-up was performed and cervical lymphadenopathy completely resolved.

## 3. Discussion

Taiwan is an endemic area of TB with an intermediate burden. With the implementation of the directly observed treatment strategy (DOTS) program, the mortality and new case decreased in recent years.^[6,7]^ Nowadays, more than 85% of TB cases could be cured. However, adverse effects including DILI, drug eruptions, gastrointestinal or neurological disorders dampened the completion of anti-TB medications, in which DILI accounts for at least 11% of discontinuation of anti-TB drugs.^[8]^

Among the first-line anti-TB agents, PZA is the most common causative drug for DILI and usually develops a hepatocellular liver injury. Previous study by Shu et al demonstrated that the incidence of PZA-related DILI was 3.71 per 100 patient-month, which was more common than the incidence of INH- or RMP-related DILI.^[5]^ Previous study demonstrated that those receiving a PZA-containing regimen had a higher risk of developing DILI.^[9]^ Durand et al described 2 different patterns of DILI in patients receiving anti-TB medication, which a PZA-containing regimen cause a delayed increase of AST and ALT 4 to 8 weeks after initiation of anti-TB therapy. A delayed onset of DILI is a poor prognostic factor during TB treatment, which could lead to liver transplantation or death.^[10,11]^ A longer half-life compared with INH and RMP might be attributed to this delayed and prolonged DILI.^[1,5]^ However, the mechanism of PZA-related DILI remained unclear. The toxicity of PZA was considered as dose-dependent, which previous studies revealed a high dose of 40 to 50 mg/kg and a longer drug exposure were associated with higher risk of developing DILI.^[1,5,10,11]^ Previous clinical trial had demonstrated that a higher dose of RMP and PZA treatment might shorten the treatment course, instead a prolonged course. Nevertheless, the optimal dose and duration remained debating.^[12,13]^ Our case highlighted that a prolonged and severe DILI still could take place under a standard dose of PZA.

As previous studies described, PZA-related DILI usually resolved within 4 weeks after discontinuation of PZA.^[14,15]^ Our patient presented a prolonged hepatocellular liver injury up to 4 months after discontinuation of a standard dose (25 mg/kg) of PZA treatment, which was rarely reported. Previous cohort study by Ichai P highlighted that INH and PZA can cause serious damage to liver function even after anti-TB drugs were terminated, in which a 45-year-old man with chronic hepatitis C and pulmonary TB died due to DILI 2 months after anti-TB medication interruption.^[16]^

In addition to an older age, the patient did not present previously known risk factor associated with DILI such as malnutrition, alcoholism or concurrent viral infection. NAT2 slow acetylator phenotype was accidentally found during workup. NAT2 is the dominant enzyme of INH metabolism, including deactivation, bioactivation and detoxification. Patients with slow acetylator phenotype had a higher risk of INH-related hepatotoxicity than rapid acetylators.^[2]^ In our case, INH was administered only for 7 days. PZA-related hepatoxicity was more likely to contribute his prolonged abnormal liver function. However, previous study showed that slow acetylator phenotype is also associated with higher incidence of PZA-related hepatitis through unknown mechanisms, in which the odds ratio of slow acetylators developing PZA-induced hepatitis compared to rapid acetylators was 3.28 (95% confidence interval 1.53‐7.06).^[17]^ Further investigation is therefore needed.

There are several limitations in this study. First, drug level including INH and PZA were not performed, which could be the direct evidence suggesting DILI. Second, we did not perform other mechanism interfering the development of DILI such as the genotype of CYP2E1.

In conclusion, our case highlights that PZA-related DILI could be delayed-onset and prolonged, and NAT2 slow acetylator phenotype is a possible risk factor which needs further investigation. Regular monitoring liver enzymes under clinical setting warrants to prevent fatal adverse effect and treatment failure.^[18]^

## Acknowledgments

The authors would like to thank Dr Chin-Chung Shu, Department of Internal Medicine, National Taiwan University Hospital for providing the tests of NAT2 SNPs of this patient.

## Author contributions

**Investigation:** Yeh-Chin Wang, Kai-Hsiang Chen, Yen-Lin Chen.

**Methodology:** Shu-Wen Lin.

**Writing (original draft):** Yeh-Chin Wang.

**Writing (review & editing):** Wang-Da Liu, Jann-Tay Wang, Chien-Ching Hung.
